# Beyond Repression: ArsR Functions as a Global Activator of Metabolic and Redox Responses in *Escherichia coli*

**DOI:** 10.3390/proteomes14010001

**Published:** 2026-01-04

**Authors:** Brett Sather, James Larson, Kian Hutt Vater, Jade Westrum, Timothy R. McDermott, Brian Bothner

**Affiliations:** 1 Department of Chemistry and Biochemistry, Montana State University, Bozeman, MT 59717, USA; 2 Department of Land Resources and Environmental Science, Montana State University, Bozeman, MT 59717, USA; timmcder@montana.edu

**Keywords:** ArsR regulation, arsenic stress response, *Escherichia coli* proteome, Ars operon expression, proteome stress response, differential proteome analysis, mass spectrometry-based proteomic analysis, proteomic pathway analysis

## Abstract

Background: The arsenic-responsive repressor, ArsR, has long been understood as a canonical regulator of the *arsRBC* operon, which confers resistance to arsenic stress. However, recent studies suggest a broader regulatory scope for ArsR. Here, we investigated the proteomic landscape of *Escherichia coli* strains with and without ArsR to elucidate ArsR as an activator in both non-stressing and arsenic-stressing conditions. Methods: Using mass-spectrometry-based shotgun proteomics and statistical analyses, we characterized the differential abundance of proteins across AW3110 (Δ*arsRBC*), AW3110 complemented with *arsR*, and wild-type K-12 strains under control and arsenite-stressed conditions. Results: Our study shows that ArsR influences proteomic networks beyond the *ars* operon, integrating metabolic and redox responses crucial for cellular adaptation and survival. This suggests that ArsR has a significant role in gut microbiome metabolomic profiles in response to arsenite. Proteins involved in alanine, lactaldehyde, arginine, thioredoxin, and proline pathways were significantly elevated in strains where ArsR was detected, both with and without arsenite. We identified proteins exhibiting an “ArsR-dependent” activation pattern, highlighting ArsR’s potential role in redox balance and energy metabolism. Conclusions: These findings challenge the classical view of ArsR as a repressor and position it as a pleiotropic regulator, including broad activation.

## 1. Introduction

The *ars* operon plays a crucial role in managing arsenic toxicity, a condition that disrupts cellular processes and threatens survival. In *Escherichia coli,* the canonical *arsRBC* operon encodes a coordinated tolerance system whereby ArsR, a transcriptional repressor, senses intracellular arsenite (As(III)) [1]. ArsR is a founding member of the SmtB/ArsR family of transcriptional repressors [2,3,4,5]. It targets a helix-turn-helix DNA-binding domain of the regulated gene(s), which then blocks transcription. Upon binding its ligand, As(III), ArsR undergoes a conformational change that releases it from the DNA operator, thereby allowing access by RNA polymerase to transcribe the *arsRBC* locus [6]. ArsC, an arsenate (As(V)) reductase, converts As(V) to As(III), which is then removed from the cell by the efflux pump ArsB [7]. Separate from ArsR regulation but integral to the process, reducing equivalents for ArsC function are provided by glutathione production [8] while working in concert with glutaredoxin A (GrxA) and glutaredoxin B (GrxB) [9]. All these proteins work harmoniously through As(V) reduction to As(III) and subsequent As(III) efflux, thereby minimizing damage to cellular functions.

Microbial arsenic resistance is not universally limited to a simple circuit. In contrast to *E. coli*, which has a single *ars*RBC operon, other organisms, such as *Agrobacterium tumefaciens*, harbor multiple *ArsR* proteins [10]. In our previous research, we challenged the traditional role of ArsR when we revealed that the four *arsR* genes (*arsR1-4*) in *A. tumefaciens* have a complex interconnected regulatory architecture that extends beyond arsenic resistance genes and includes pathways related to phosphate metabolism, motility, and central carbon flux [11]. Additionally, we showed a regulatory hierarchy among the four ArsR proteins in *A. tumefaciens* whereby regulatory control was exerted over one another. For example, ArsR1 represses *arsR4*, and ArsR4 activates *arsR2*, etc. One particularly interesting finding was that every single ArsR protein regulated at least 1, if not more, metal homeostasis-related proteins in that organism.

Based upon these findings, and the central role of metalloenzymes [12], we initially explored metalloproteome (complete set of metalloproteins in a given organism) differences in a biological system containing only one *arsR* gene, and logically a less-sophisticated ArsR regulatory network. We worked with *E. coli* strains having varied *ars* gene composition, exposing them to high but sub-lethal As(III) stress [13]. This research confirmed ArsR to have a broader regulatory effect on the metalloproteome and metal homeostasis. The presence of ArsR influenced the balance of metals such as iron, copper, and nickel and additionally enhanced resistance to As(III). Proteins involved in the synthesis of a molybdenum cofactor and iron sulfur [Fe-S] clusters were upregulated, indicating that these pathways may be vital for managing stress caused by As(III) exposure. Additionally, an increase in TCA cycle enzyme activity was observed when ArsR was present [13]. This may contribute to promoting pathways involved in alleviating stress on cells. By comparing across the chosen *E. coli* strains and treatments, a distinct pattern emerged: several metalloproteins and metabolic pathways exhibited elevated abundance only in conditions where ArsR was present. These results suggested that ArsR’s regulatory influence extended well beyond its classical role controlling *arsRBC* in response to As(III) and into the metalloproteome.

The above research contributes to microbe–arsenic interaction research over the past approximately three decades that has focused on illustrating how microbial arsenic oxidation, reduction, and (de)methylation transformations can influence the relative toxicity and mobility of arsenic in a range of environments (e.g., soils, aqueous, geothermal, etc.). Recent discoveries regarding the importance of the gut microbiome have shifted attention to the human intestinal tract environment. Upon arsenic ingestion (primarily drinking water), the gastrointestinal tract microbiome is positioned to immediately begin acting. The broad range of possible microbial arsenic biotransformations [14] prompts questions regarding the microbiome’s role with regard to mitigating or indeed exacerbating human health problems associated with arsenic exposure. Surveys of human gut microbial metagenomes have revealed the presence of many of these same *ars* genes and encoded functions, including *arsR.* Accordingly, it is important to understand how ArsR regulates microbiome gene expression in this important environment. Work with the gut-relevant *E. coli* [15] advances these efforts.

Here, we sought to illuminate the innate regulatory roles of ArsR to the global proteome in *E. coli* in both arsenic-stressing and non-stressing conditions, which we have previously explored solely in the context of metalloproteins. We utilized an *E. coli* strain that possesses and constitutively expresses only one *arsR* gene and thus allows for a rather focused assessment of ArsR on proteomic perturbations. *E. coli* is highly relevant to the human gut microbiome environment, which is now known to be critical to human health and well-being [16]. Near-isogenic strains of *E. coli* were chosen with the goal of observing differences in the proteome responses in the absence or presence of the *arsRBC* operon, as well as under and without As(III) stress. The three strains selected included (i) AW3110, wherein the entire arsRBC operon is deleted and replaced by a chloramphenicol cassette (∆*arsRBC*); (ii) AW3110 carrying a complementing extrachromosomal *arsR* (referred to here as the *arsR*-complement); and (iii) the *E. coli* wildtype strain K-12 MG-1655. K-12 was included to enable us to assess potential effects of ArsR copy number, i.e., a single genome copy of *arsR* in K-12 vs. *arsR* carried on a low-copy-number plasmid in AW3110 (*arsR*-complement). We explored the potential expanded scope of ArsR influence by a global proteomic analysis of the above *E. coli* strains. By comparing proteome-wide abundance patterns, we aimed to identify proteins and pathways that exhibit ArsR-dependent regulation, independent of direct As(III) interaction. This broader investigation, detailed in the sections that follow, reveals novel functional dimensions of ArsR as a potential activator and systems-level regulator.

## 2. Materials and Methods

### 2.1. Plasmid Design, Cloning, and Transformation of arsR into AW3110

The full coding sequence of the *arsR* gene of *E. coli* K-12 W3110 (NP_417958), with its endogenous promoter but without the ArsR binding domain, was amplified by PCR using primers ArsR-F (AAATTAATTAATATTACCTTCCTCT-GCACTTAC) and ArsR-R (AAACCTAGGTTAACTGCAAATGTTCTTACTGT). PCR products were inserted into the pCR2.1 TOPO TA vector (Invitrogen™, Waltham, MA, USA) following the manufacturer’s instructions, generating the *arsR*-complemented AW3110 strain. Successful transformation was confirmed via GENEWIZ’s Sanger sequencing service (Azenta Life Sciences, Burlington, MA, USA).

### 2.2. E. coli Strains and Growth Conditions

In addition to the *arsR*-complement, *E. coli* strains K-12 MG1655 (wild-type) and AW3110 (Δ*arsRBC* mutant with a chloramphenicol resistance marker) were generously provided by Dr. Barry Rosen [17]. Cultures of *E. coli* were grown in 200 mL of LB broth under batch conditions at 37 °C. LB was freshly prepared using tryptone (MilliporeSigma, Burlington, MA, USA), yeast extract (Thermo Scientific, Waltham, MA, USA), and sodium chloride (Fisher Scientific, Waltham, MA, USA).

Cultures were inoculated with 2 mL of overnight-grown cells, and growth was monitored using OD_600_. Three replicates were used for each strain and condition. AW3110 and the *arsR*-complement AW3110 were cultured with chloramphenicol and kanamycin selection, respectively. As(III), given as sodium (meta)arsenite (Sigma-Aldrich, St. Louis, MO, USA), was added 2 h after inoculation. Based upon prior work, K-12 was stressed with 1 mM As(III) while AW3110 and the *arsR*-complement were stressed with 100 µM As(III) to elicit similar As(III) stress responses that are sub-lethal. Collectively, these are referred to as (H) arsenic stress. As an additional control, K-12 *E. coli* was also given 100 µM As(III), referred to as low (L) arsenic stress. Cultures were harvested at two hours post-As(III) treatment, which was 4 h post-inoculation (mid-log phase) for all samples. A 50 mL aliquot of each culture was harvested by centrifugation (4000× *g*, 10 min, 4 °C) and stored at −80 °C. Growth data can be found in our previous work [13].

### 2.3. Cell Counts and Cell Size

Cell counts for K12 *E. coli* were assessed for the high As(III) condition (1 mM As(III)) and the control condition (0 mM As(III)). Each culture condition was grown as described above. OD600 measurements were taken 4 h after culture inoculations.

At this time, liquid cultures of K12 *E. coli* (100 µL each) were also diluted (PBS, 2% FBS, 0.1% Sodium Azide) and stained for 30 min at 4 °C in the dark with SYBR Green (Invitrogen DNA Safe Gel Stain #S33102, final dilution 1:1:7750) in a total volume of 300 µL. After incubation, 10 µL of Invitrogen CountBright Plus Absolute Counting Beads (5 µm, 1.06 × 10^4^ beads/10 µL; Invitrogen, Waltham, MA, USA) or Sizing Beads (0.1 µm, 0.2 µm, 0.5 µm, 1 µm, 2 µm; Invitrogen) were added to each sample. Samples were analyzed in MSU’s Cellular Analysis Core (RRID:SCR_026328) on a BioRad ZE5™ Cell Analyzer (BioRad, Hercules, CA, USA), and data analysis was performed using FlowJo software v10.10.1 (Becton Dickinson, Franklin Lakes, NJ, USA). A linear standard curve was generated to determine bacterial sizes based on the sizing beads. Cell counts for samples used in shotgun proteomics were determined using the control and As(III) stressed K-12 *E. coli* flow cytometry results.

### 2.4. Isolation of Proteomes

Cell pellets were washed three times by gentle agitation in 5 mL of lysis buffer (200 mM ammonium acetate, pH 7). Between washes, cells were spun at 1500× *g* for 5 min at 4 °C. Following the washes, cells were resuspended in 800 µL of lysis buffer and lysed using Matrix E beads (MPBio, Irvine, CA, USA) in a FastPrep-24 5G homogenizer (MPBio) operating at 6.0 m/s for 30 s. Cell lysate were centrifuged (18,000× *g*, 20 min, 4 °C) to pellet insoluble debris, and the supernatant was collected. Protein concentration was then measured using the Bradford method [13].

### 2.5. Proteomic Data Acquisition

Soluble lysates were standardized to protein concentration. The proteomes (100 µg) were chemically reduced and alkylated and subjected to a chloroform/methanol-based cleanup before digestion with sequencing-grade porcine trypsin for shotgun proteomics (Promega, Madison, WI, USA). Digestions were conducted at the IDeA National Resource for Quantitative Proteomics (University of Arkansas, Little Rock, AS, USA). The peptides were separated using 1 µg of digest with reverse-phase chromatography (XSelect CSH C18, 2.5 µm, Waters, Milford, MA, USA) on a 150 × 0.075 mm column fitted to an UltiMate 3000 RSLCnano system (Thermo Scientific). A 60-minute gradient (from 98:2 to 65:35, solvent A/B, where A = 0.1% formic acid, 0.5% acetonitrile, B = 0.1% formic acid, 99.9% acetonitrile) was applied for elution. Ionization was performed using electrospray (2.4 kV), and data were acquired with an Orbitrap Eclipse Tribrid mass spectrometer (Thermo). MS1 scans were collected in profile mode at a resolution of 120,000 across a 375–1200 *m*/*z* range. Peptides were fragmented by HCD and analyzed in the ion trap using centroid mode with normalized collision energy set to 30%. Protein identification was carried out via MaxQuant [18] (Max Planck Institute, Munich, Germany) using a parent ion tolerance of 3 ppm and fragment ion tolerance of 0.5 Da. Scaffold Q+S (v5.0.1, Proteome Software) was used to confirm identifications. Proteins were accepted if supported by at least two peptides and <1.0% FDR. Probabilities were assigned via the Protein Prophet algorithm [19]. For further information on the methods used, please see the supplemental methods. Raw protein intensities, UniProt accession numbers, and UniProt protein names generated from the acquisition have been provided in the Supplemental Information (Supplemental Table S1).

### 2.6. Statistical Analysis of Proteomic Data

Protein datasets were processed using MetaboAnalyst version 6.0 (https://www.metaboanalyst.ca/ URL accessed 1 November 2024) [20]. Data normalization parameters included sum normalization, autoscale (mean-centered and divided by the standard deviation of each variable), and log_10_ transformation. Two separate one-way ANOVAs were applied (FDR thresholds of 0.05 and 0.001), followed by Tukey’s HSD post hoc tests with cutoffs of 0.001 to isolate proteins with statistically significant differences across conditions. A complete list of all proteins that are significantly different at an FDR < 0.001 can be found in Supplemental Table S2. Boxplots of normalized abundances were created for significant proteins. PCA and K-means clustering were conducted across all treatment groups and on subsets experiencing elevated As(III) stress.

After determining the proteins that passed ANOVA, we next examined whether their abundance changes followed a consistent “ArsR-dependent” pattern across strains. A protein was retained if it was more abundant in the ArsR-complemented strain than in the Δ*arsRBC* mutant (AW3110) and if this same trend was observed in the wild-type K-12 strain. This comparison was made independently under both control and arsenite-treated conditions. In essence, we kept proteins that consistently followed the pattern *ArsR present* > *ArsR absent* in at least one condition, ensuring that changes reflected ArsR’s regulatory influence rather than random variation. (Formerly, the inclusion rule corresponded to either (arsR_C > AW_C and K-12_C > AW_C) or (arsR_AsIII > AW_AsIII and K-12_L/H_AsIII > AW_AsIII). To illustrate some specific comparison of As(III) and ArsR effects, paired t-tests were used to assess statistical differences.

Non-detected proteins were coded as missing values (shown as “0” in the tables) and handled by the statistical pipeline. All groups were analyzed in triplicate. Proteins with only sporadic replicate detection are retained in the dataset for completeness, but the associated high variance prevents them from driving significant results unless effect sizes are large.

### 2.7. Visualization of Transcription Regulation Networks

The genes of proteins that demonstrated a pattern of interest (increased abundance when ArsR was present) were manually examined in the online databases RegulonDB [21] and EcoCyc [22] to identify respective transcription factors. The regulation of the identified transcription factors was explored in the same fashion. The regulatory networks were then visualized using Flourish Studio.

## 3. Results and Discussion

### 3.1. Proteome Response to Arsenite Stress

To elucidate the full impact of ArsR on the *E. coli* proteome, a three-way comparison with two primary treatment conditions was effectuated. The primary comparison was between AW3110 and the *arsR*-complement, while K-12 was used as a wild-type reference. The three *E. coli* cell types were cultured with and without arsenite (As(III)) to compare proteomic responses and determine ArsR-dependent regulatory effects. Comparison of *arsR*-complement with AW3110 should provide ArsR-specific changes in the absence and presence of As(III), as *arsR* should be constitutively expressed. Cell counts were determined via flow cytometry using K-12 control and high arsenic-stressed cells as a reference (Supplemental Table S3). In our samples, we identified 1814 proteins using shotgun proteomics (Supplemental Table S1). ANOVA analysis showed that 889 proteins were significantly different in abundance in at least one group using an FDR-corrected *p*-value threshold of 0.001 (Figure S1). The number of significantly changed proteins highlights the broad impact of ArsR and the global impact of As(III) induced stress.

**Figure 1:**
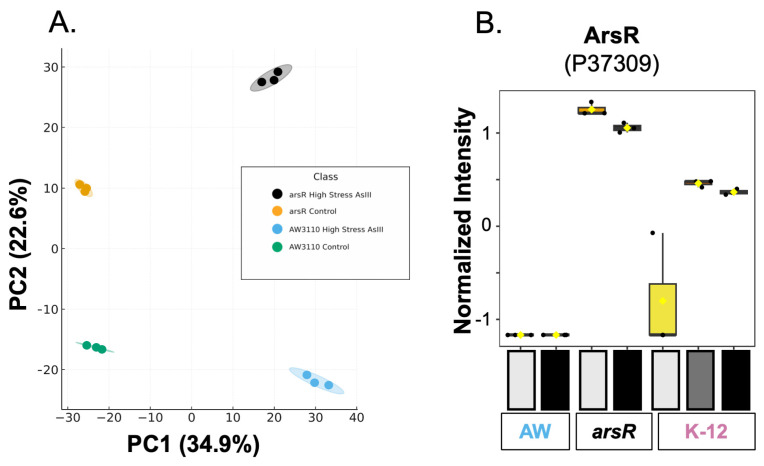
Principal component analysis and ArsR abundance of treatment groups. (**A**) Principal component analysis (PCA) of proteome profiles for *E. coli* strains and treatments. Points are biological replicates; shaded ovals show 95% confidence ellipses. Colors: black = arsR High As(III) Stress, orange = arsR Control, light blue = AW3110 High As(III) Stress, green = AW3110 Control. K-12 samples were excluded. Data are PCA scores from autoscaled protein intensities; PC1 (34.9%) and PC2 (22.6%) jointly separate samples by ArsR and arsenite treatment. (**B**) ArsR (P37309) across three *E. coli* strains (AW3110 in blue, arsR-complement in black, and K-12 in pink) exposed to increasing arsenite levels. Arsenite levels are indicated by bar shading from light gray (control) to black (high arsenic stress for the given strain). For ArsR, the relative intensity is also shown to provide context for how much ArsR is present relative to the other groups in the *arsR* complement groups.

Following the ANOVA analysis, a principal component analysis (PCA) was implemented to visualize the differences between the groups. In our initial PCA plot (Figure 1A), we excluded K-12 strains to clearly observe proteomic differences driving separation between the near-isogenic strains with (*arsR*-complement) and without (AW3110) ArsR. PC1 and PC2 explained 34.9% and 22.6% of the variance, respectively, and replicates were clustered tightly, indicating low ingroup variance and high reproducibility. PC1 primarily reflected the As(III) treatment effect as both strains shifted from negative PC1 (controls) to strongly positive PC1 (high As(III) stress). However, PC2 was separated by the presence or absence of ArsR, whereby ArsR-expressing samples loaded high on PC2, and AW3110 samples loaded low under both control and As(III) conditions. Our PCA suggests that ArsR is affecting the global proteome both in the absence and presence of arsenite.

PCA was also performed on all groups, where (PC) 1 and 2 explained 25.2 and 21.9% of the variance, respectively (Supplemental Figure S2). The biological replicates grouped tightly, indicating high within-group consistency. AW3110 and the *arsR* complement control groups clustered closely together, while their respective As(III)-treated samples also grouped similarly along the PC 1 axis, suggesting similar proteomic responses between these strains. K-12 strain groups exhibited a different response (Supplemental Figure S2) separated from the other two strains primarily by the PC 2 axis in all treatment conditions. Some separation can also be observed along the PC 1 axis between K-12 group conditions and the *arsR* complement, as well as AW3110 groups. However, controls for AW3110 and the *arsR* complement aligned with the K-12 strain control and low As(III) stress groups along the PC1 axis. The same alignment is observed between AW3110 and the *arsR* complement under high As(III) stress and the K-12 high As(III) stress group along the PC 1 axis. Strain differences were therefore separated by variations found within the PC 2 axis, and treatment conditions were separated by variations dictated by the PC 1 axis.

We then employed K-means clustering on the PCA-transformed data to group samples based on overall similarity in their normalized protein abundance profiles. K-means was selected because it provides an unsupervised, geometry-based partitioning that complements PCA by quantitatively assigning samples to discrete clusters according to Euclidean distance in principal component space. This approach is well-suited for moderate-sized datasets, such as ours, where sample groups are already linearly separable along the first few PCs, and allows direct comparison of cluster membership to biological conditions. Three clusters were detected: Cluster 1 contained all groups exposed to the highest respective As(III) stress, indicating a distinct separation along PC 1. Cluster 2 contained K-12 samples that were either untreated or subjected to low As(III) stress, while Cluster 3 included As(III) stressed samples from AW3110 and the *arsR* complemented strain. The ability of K-means to distinguish between high and low/no arsenite stress, as well as across different genetic backgrounds, provides additional evidence of the distinct biological impact of high As(III) stress across conditions.

To gain insight into the differences between these samples, we began to look at the abundance of individual proteins. We started by confirming our experimental design was working correctly by identifying the abundance of ArsR across sample groups and conditions. As expected, ArsR (P37309) was constitutively expressed in the *arsR*-complement samples, not observed in the AW3110 group, and only background expression (detected in a single replicate at ~3 orders of magnitude lower) in the K-12 control group (Figure 1B). The abundance of ArsR increased in K-12 treatment groups, consistent with the known transcriptional control of *arsR*. The greatest abundance of ArsR was observed for K12 and the *arsR* complement (Figure 1B). Interestingly, when we searched using directional contrasts from the ANOVA post hoc analysis and manually confirmed the ANOVA results of each protein, we observed a unique pattern in our data. As ArsR increased in abundance (AW3110 → K12 control → K12 high As(III) stress → K12 low As(III) stress → *arsR* complement high As(III) stress → *arsR* complement control), specific groups of proteins also increased in abundance. This became a pattern of interest with respect to protein abundance and is defined as proteins with increased abundance associated with strains containing ArsR, either produced through expression of *arsR* in the presence of arsenite (K-12 strains) or induced, as in the case of our *arsR-complemented* strain. This pattern shows that ArsR may be functioning either directly or indirectly as an activator separate from its well-characterized repressor action and is consistent with observations in *A. tumefaciens* [10,23,24], and encodes multiple *arsRs,* so it would be logical that activation may be unique to this microbe. This novel activating role of ArsR in *E. coli* impacted a number of metabolic pathways, including pyruvate, arginine, glutamate, proline, and redoxin metabolisms (Supplemental Table S4).

### 3.2. Pathways Associated with Pyruvate Metabolism

#### 3.2.1. Alanine Degradation

One regulatory pattern of interest included DadX (alanine racemase 2, P29012) and DadA (amino acid dehydrogenase, P0A6J5), which are involved in the alanine degradation pathway by converting alanine into pyruvate via racemization and oxidative deamination of alanine. As a general pattern, increased relative abundances of DadX and DadA reflected the presence, though not necessarily abundance, of ArsR (Supplemental Figure S3). For both As(III) and control cells, DadX and DadA abundance in AW3110 were significantly lower than in K-12 and the *arsR*-complement groups. However, DadX in As(III)-treated AW3110 was at least an order of magnitude greater than in control cells, implying *DadX* expression is not directly tied to ArsR at the transcriptional level. For both AW3110 and the *arsR* complement, DadA abundance was significantly lower in As(III)-treated cells, and likewise was lower at the highest As(III)-treated K-12 cells. DadX showed minimal, reduced abundance in high As(III)-stressed K-12. Alanine catabolism may play a role in maintaining intracellular pyruvate pools, critical for metabolic flexibility under stress. Spatial and temporal regulation of alanine utilization in *E. coli* biofilms was demonstrated that alanine is catabolized to pyruvate via DadX and DadA, supporting growth and viability in nutrient-limited conditions [23]. Furthermore, modulation of central carbon flux through alanine degradation has been shown to impact pyruvate availability and stress resilience, with pyruvate supplementation rescuing growth in alanine-auxotrophic strains [24]. These findings suggest that differential regulation of DadX and DadA by ArsR may contribute to an adaptive strategy to fine-tune pyruvate availability regardless of arsenic.

#### 3.2.2. Lactaldehyde Degradation

**Figure 2:**
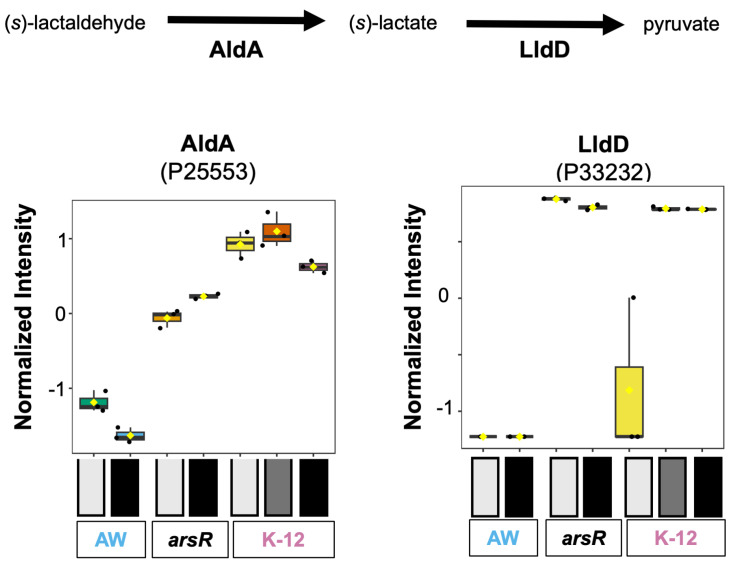
Lactaldehyde degradation is upregulated in the ArsR-expressing strains. Normalized intensities of lactaldehyde degradation member proteins, AldA (P25553) and LldD (P33232), expressed by the different *E. coli* strains in each condition are shown in the box and whisker plots. The strains are represented by either AW for AW3110, *arsR* for the *arsR* complemented strain, or K-12. Increasing darkness below the plots indicates the severity of As^III^ stress. For each strain, the no-As^III^ control is light gray, the low-As^III^ stress is gray, and the high-As^III^ stress is black.

Pyruvate can be produced through the lactaldehyde degradation pathway. The enzymes involved with this, AldA and LldD, exhibited the abundance pattern of interest (Figure 2). Lactaldehyde degradation converts (*S*)-lactaldehyde into pyruvate using aldehyde dehydrogenase A (AldA) and lactate dehydrogenase (LIdD) with lactate as an intermediate metabolite. The abundance of AldA (P25553) was substantially elevated in the K-12 strain groups compared with AW3110 and the *arsR*-complemented strain. AldA contributes to several additional metabolic pathways as it catalyzes the irreversible oxidation of lactaldehyde to lactate, but also acts on glycolaldehyde and glyceraldehyde [25]. Therefore, AldA abundance, given its diverse functionality, does not necessarily predict meaningful involvement with *arsR* from this data, but the presence of ArsR clearly increases the protein’s abundance. However, when tied to the lactaldehyde degradation pathway and subsequent LldD abundance, a compelling observation is revealed, indicating a potential ArsR regulatory role.

An abundance of lactate dehydrogenase (P33232, LIdD) was strictly tied to ArsR. Specifically, LldD is higher in conditions where ArsR is being expressed (Figure 1B and Figure 2). LldD contributes to lactaldehyde degradation indirectly by converting lactate, produced from lactaldehyde via AldA, into pyruvate, with its expression regulated by LldR and carbon source availability, integrating the pathway into aerobic energy metabolism through the TCA cycle [26]. While direct regulation of LldR is not provable here, there does appear to be a strong correlation between ArsR and LldD abundance. These findings provide new evidence that *E. coli* responds to As(III)-induced stress by altering its metabolic pathways, including those associated with pyruvate generation, to maintain energy homeostasis and enhance survival under adverse conditions.

#### 3.2.3. Arginine Degradation II (AST Pathway)

**Figure 3:**
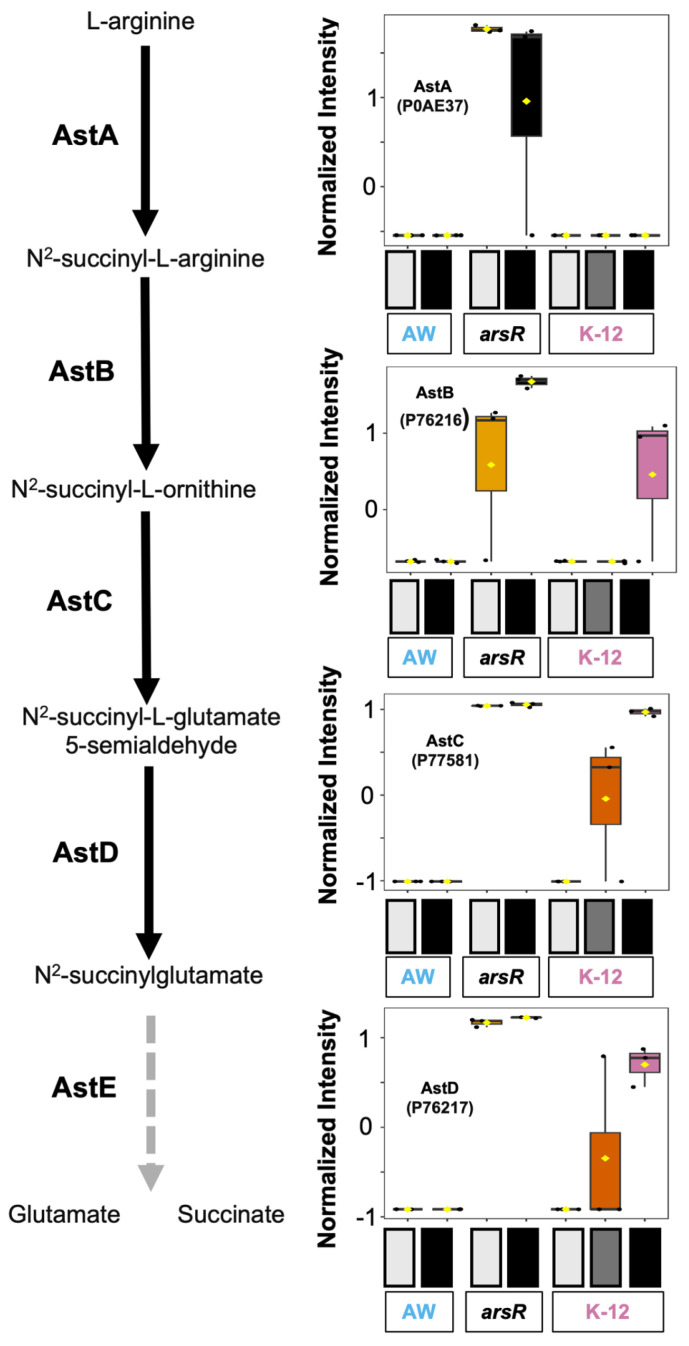
Regulation of the arginine succinyltransferase (AST) pathway under arsenic exposure. This figure illustrates the expression trends of four enzymes (AstA, AstB, AstC, and AstD) that mediate the conversion of arginine to either glutamate or succinate. AstE was not found in the data, but is represented where it exists in the pathway with a dotted gray arrow. Corresponding ANOVA-normalized boxplots visualize relative protein levels under control (light gray), low-arsenic (gray), and high-arsenic (black) treatments. The strains are represented by either AW for AW3110, *arsR* for the *arsR* complemented strain, or K-12.

The arginine degradation (AST pathway) feeds into the TCA cycle [27]. Arginine *N*-succinyltransferase (AstA, P0AE37) initiates this pathway, continuing through AstB (Succinyl ornithine transaminase, P76216), AstC (Succinylornithine transaminase, P77581), AstD (Aldehyde dehydrogenase, P76217), and then AstE (succinylglutamate desuccinylase, P76215), which yields glutamate and succinate. We identified all these enzymes, except for AstE, in our dataset (Figure 3). All the enzymes identified in this pathway had the expression pattern of interest, except for AstA, which had a slightly different pattern in K-12, where it was below detection across conditions. Despite this, AstA was higher in the *arsR*-complement groups when compared to AW3110 groups (*p* = 0.006). Interestingly, AstB, AstC, and AstD were strongly upregulated in both the *arsR*-complement control and As(III)-treatment groups when compared to the respective AW3110 groups (*p*-values = 0.004, <0.0001, and <0.0001, respectively). Additionally, K-12 groups treated with both low- and high-stress arsenic were higher in abundance than the K-12 control and AW3110. This data suggests that the presence of ArsR plays a significant role in the expression of the AST pathway, which in some cases is independent of As(III).

We draw attention to the significant upregulation of all Ast proteins in *arsR*-complemented AW3110 as compared to AW3110 (*p*-values < 0.0001 to 0.006) (Figure 3). These upregulation events included cultures absent of As(III), which indicates ArsR is behaving as an activator and serves as another example of ArsR having functions other than as a well-documented repressor, and supports what we have previously reported for ArsR proteins in *A. tumefaciens* [28].

We also note that we encountered some unanticipated instances where an Ast protein of interest was not detected in one of three replicates in various strain/treatment combinations, resulting in large apparent variation (Figure 3). This included +/− As(III) comparisons of AstA and AstB in AW3110 (arsR), although it did not change the statistical conclusion that As(III) exposure did not influence their expression in this strain/As(III) combination. AstB, AstC, and AstD were similarly variable in As(III) treatments of K12, but again, it did not alter the conclusion that expression of these proteins was enhanced when K12 was treated with As(III) (*p*-values = 0.079, 0.016, and 0.031, respectively; ANOVA, Tukey HSD).

The observed increase in AST pathway enzymes abundance in *arsR*-expressing strains aligns with previous findings showing upregulation of stress-responsive metabolic systems during heavy metal exposure. In a recent transcriptomics study, it was shown that arginine biosynthesis and uptake were increased in response to palladium exposure in *E. coli* [29]. The authors found that genes associated with arginine utilization were consistently upregulated, indicating a potential role for arginine metabolism in mitigating the effects of heavy metal stress, which correlates with the findings of our report. Additionally, the authors used M63 minimal media, which is devoid of arginine, and yet similar responses were observed to the LB media used in our study. The AST pathway’s function in facilitating arginine degradation into glutamate and succinate, a route that can replenish central metabolic intermediates, suggests a metabolic advantage under nutrient or redox stress. The strong association between strains with high ArsR abundance and higher abundance of AST proteins supports the role of ArsR as more than just a repressor for *arsRBC*.

### 3.3. Protein-Facilitated Fates of Glutamate

Beyond playing crucial roles as an essential amino acid and as a primary substrate in a plethora of metabolic processes, glutamate is important in stress responses for cells [30]. Several glutamate-related pathways were identified as having the abundance pattern of interest, where increased abundance in the proteome of these pathways was associated with ArsR-producing strains. These related pathways include the thioredoxin-based stress-response system and proline biosynthesis. Below, we discuss these pathways and other observations in related glutathione biosynthesis and glutaredoxin proteins.

#### 3.3.1. Trx, Gsh, and Grx Protein Systems Related to Glutamate

**Figure 4:**
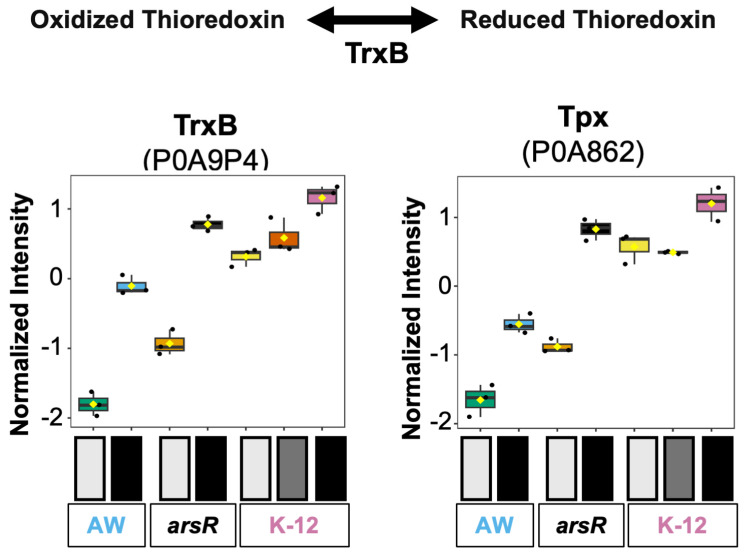
Differential expression of thioredoxin proteins in response to arsenite exposure across *E. coli* strains. Normalized protein levels of enzymes involved in thiol–redox homeostasis include thioredoxin reductase TrxB (P0A9P4) and thiol peroxidase Tpx (P0A862). On the right, ANOVA-normalized expression levels are displayed by condition: light gray for control, gray for low arsenic, and black for high arsenic exposure. The strains are represented by either AW for AW3110, *arsR* for the *arsR* complemented strain, or K-12.

Thioredoxin and glutaredoxin-driven systems are critical for redox balance under oxidative stress [31], utilizing thiol chemistry to mitigate damage and maintain cellular function [32]. These systems are known users of glutathione [32], linking established glutamate metabolism to stress-responsive redox buffering. TrxB (thioredoxin reductase, P0A9P4) is a central component of the thioredoxin system, facilitating electron transfer to thioredoxins and maintaining them in a reduced state for supporting electron donations to proteins involved in mitigating oxidative stress [33]. In our dataset, TrxB abundance followed the ArsR-related response. Under high As(III) stress, the *arsR*-complement strain had TrxB levels comparable to those of K-12 strains under both low and high As(III) stress (Figure 4). While TrxB is present in AW3110, its abundance under As(III) stress was markedly lower than in *arsR*-containing strains (K-12 and the *arsR* complement), implying potential regulation or co-regulation by ArsR-related pathways that bolster expression of TrxB in response to As(III) stress. Tpx (Thiol peroxidase, P0A862), another thioredoxin-linked antioxidant enzyme, displayed an abundance profile that was very similar to that of TrxB (Figure 4). Tpx in *E. coli* has been shown to reduce hydroperoxides [34], and Tpx in other organisms has been shown to work on peroxynitrites [35], working in tandem with Trx to neutralize reactive oxygen species from arsenic exposure [35]. Together, this data indicates a potential ArsR-driven prioritization of thioredoxin-dependent oxidative resistance mechanisms against As(III) toxicity.

Thioredoxin activity is closely linked to glutathione biosynthesis [33], which uses glutamate to produce glutathione, a well-documented thiol-containing molecule. Glutathione has been documented with stress response mechanisms [31] and As(III) tolerance [36].

In contrast to the above patterns relating Trx and Tpx abundance to ArsR presence, we observed an inverse relationship between the abundance of glutathione biosynthesis enzymes and the presence of ArsR. GshA (glutamate–cysteine ligase, P0A6W9) catalyzes the first step of glutathione synthesis; it was most abundant in AW3110 under As(III) stress (Supplemental Figure S4), with lower expression in *arsR*-complement and K-12 strains across both As(III) treated conditions. This pattern suggests GshA may represent a compensatory response in the absence of ArsR (Supplemental Figure S4). GshB (glutathione synthetase, P04425), which completes glutathione biosynthesis, was elevated across all As(III) treated samples, including K-12 and the *arsR*-complement groups, while remaining lowest in AW3110 controls (Supplemental Figure S4). The pattern implies broad As(III)-induced upregulation of GshB, largely independent of ArsR status.

The thioredoxin and glutaredoxin systems help sustain a reduced intracellular environment by using thiol groups in concert with glutathione [37], functioning as antioxidants and electron donors in redox signaling processes that counteract reactive oxygen species [38]. Glutaredoxin members GrxA (reduced glutaredoxin 1, P68688), GrxB (reduced glutaredoxin 2, P0AC59), and GrxC (reduced glutaredoxin 3, P0AC62) are enzymes that facilitate the reduction in disulfide bonds via thiol–disulfide exchange [32]. Notably, GrxB is utilized in ArsC-dependent arsenate reduction [9]. All three Grx enzymes were found in the dataset (Supplemental Figure S5). GrxA displayed consistently elevated expression in all strains across under As(III) stress, suggesting positive regulation in response to enhanced glutathione turnover, regardless of ArsR. GrxB and GrxC showed a decrease in expression in AW3110 under high As(III) stress, but in the *arsR* complement, As(III) stress resulted in higher levels of these proteins. This implies that ArsR may be involved in the activation of these enzymes under As(III) conditions. However, in K-12 under high As(III) stress, both GrxB and GrxC decreased in abundance. Together, these results suggest GrxA serves a more critical role under As(III) stress. GrxB and GrxC exhibited higher abundance in only the *arsR*-complement strain, which may support the idea that ArsR indirectly coordinates multiple branches of thiol–redox defense. However, beyond the established *ars* operon, a complicated regulation of GrxB and GrxC may be partially regulated by a combination of As(III), ArsR abundance, but also the presence of ArsB and ArsC. Observations highlighted here show the functional role of ArsR with thioredoxin regulation, and glutaredoxin pathways in As(III)-induced oxidative stress management.

The upregulation of these enzymes also serves as internal “standards” that verify that *E. coli* was responding in a predictable fashion to As(III) stress. Oxidative stress is well known response of bacteria dealing with arsenic insult, with enzymes such as glutathione synthetase and glutathione reductase acting as keystone enzymes (Supplemental Figure S6) [39,40,41]. As such, their upregulation serves to authenticate the response of the other proteins observed in this study.

#### 3.3.2. Proline Biosynthesis

**Figure 5:**
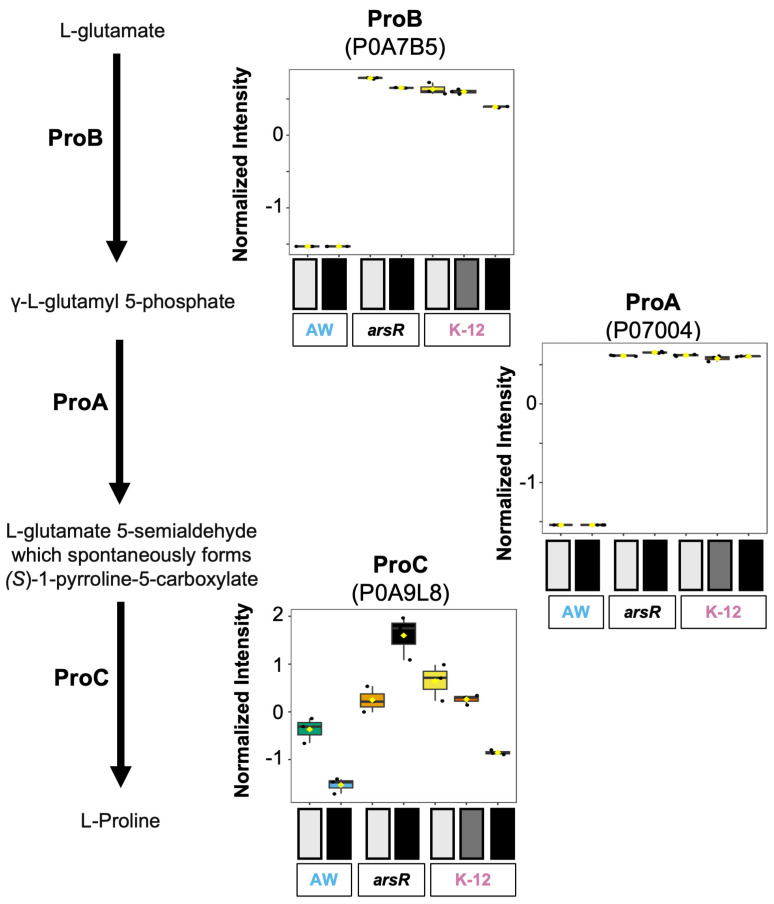
Differential regulation of L-proline biosynthesis I under arsenic stress across *E. coli* strains. Expression levels of three key enzymes involved in the proline biosynthesis pathway—ProB (P0A7B5), ProA (P07004), and ProC (P0A9L8). ANOVA-normalized intensity plots on the right illustrate protein abundance under increasing arsenic exposure, from control (light gray) to high As(III) Stress (black). The strains are represented by either AW for AW3110, *arsR* for the *arsR*-complemented strain, or K-12.

Another glutamate-dependent pathway, proline biosynthesis, exhibited protein abundance patterns consistent with an ArsR-linked regulatory pattern. This pathway converts glutamate into proline using three enzymes, including Pro(B) (glutamate 5-kinase, P0A7B5), ProA (glutamate-5-semialdehyde dehydrogenase, P07004), and ProC (pyrroline-5-carboxylate reductase, P0A9L8). All three proteins were more abundant in the *arsR*-complemented strain exposed to As(III) when compared to the AW3110 samples (Figure 5). Group comparison between the *arsR*-complemented strain and AW3110 showed an increase in abundance for ProA and ProB for the *arsR*-complemented strain. The pattern observed for ProC was less distinct, yet the *arsR*-complemented strain was higher for both the control and As (III) groups when compared to AW3110. K-12 resembled the *arsR*-complement group for ProB and ProA, but with a different trend between control and As(III) treatment groups, whereby a slight reduction in abundance was observed in the K-12 groups versus the *arsR*-complemented strain. These trends appeared to be unrelated to As(III) treatment except for ProC, where less abundance in the As (III) groups was observed in the K-12 samples in contrast to the *arsR*-complemented strain, which showed higher abundance in the As(III)-treated cells versus control. Additionally, both AW3110 groups were significantly lower in abundance for ProA and ProB compared to K-12, which mirrors the *arsR*-complement groups’ comparative abundance (Figure 5). Furthermore, in AW3110, the ProC abundance levels for As(III)-stressed versus control showed a sharp decrease. The ProC abundance for As(III) treated AW3110 was lower relative to all the other strains and As(III) treatments. Proline metabolism in *E. coli* can have significant contributions to cell health. Proline contributes, when acted upon by the flavoenzyme PutA, to enhance oxidative stress resistance by triggering a proadaptive response involving endogenous hydrogen peroxide production. This response has been shown to upregulate OxyR-regulated genes such as *katG*, *grxA*, and *trxC*, with hydroperoxidase I, which plays a key protective role [42]. These results imply that ArsR plays a role in proline generation from glutamate.

### 3.4. Uncharacterized Protein YgbM with a Unique Abundance Pattern

**Figure 6:**
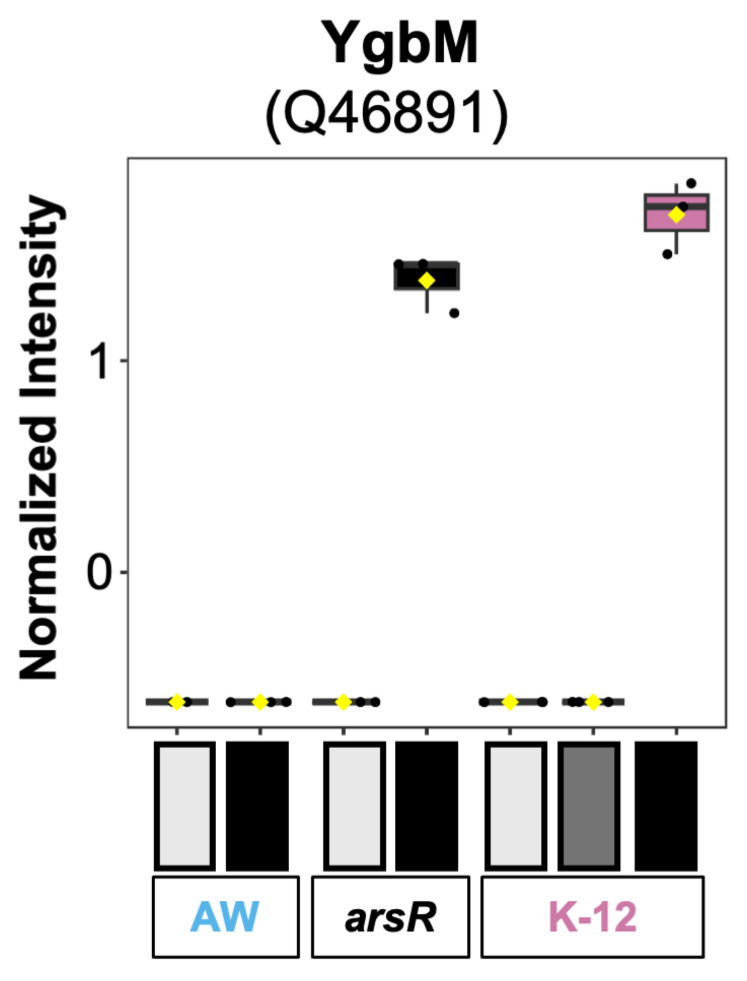
Expression profiles for YgbM from all strains and groups. Normalized protein intensity of YgbM (Q46891) across three *E. coli* strains (AW3110 in blue, arsR-complement in black, and K-12 in pink) exposed to increasing arsenite levels. Arsenite levels are indicated by bar shading from light gray (control) to black (high arsenic stress for the given strain). The strains are represented by either AW for AW3110, *arsR* for the *arsR* complemented strain, or K-12.

One of the more interesting proteins found in the dataset is YgbM (Q46891). This protein is almost completely absent in all the groups except for the *arsR*-complement cells treated with As(III) and the high-As(III)-stressed group of K-12 (Figure 6). Even the low As(III) stress K-12 group exhibited little abundance, indicating that high As(III) stress in the presence of ArsR causes differential expression of this protein. Therefore, a strong potential exists for YgbM to be upregulated in *E. coli* under As(III) stress by ArsR. Information regarding YgbM is limited, but a crystal structure has been solved [43], and a proposed enzyme function was identified as catalyzing the isomerization of 2-oxo-tetronate to 3-oxo-tetronate [44]. This may be part of a metabolic pathway involving acid sugars, where YgbM works in conjunction with YgbL, which decarboxylates 3-oxo-tetronate to produce downstream metabolites. This activity may be a part of a proposed catabolic pathway for acid sugars, where intermediates are processed into central metabolites like dihydroxyacetone phosphate (DHAP), integrating into glycolysis. The findings of this study may reveal another role and a regulatory component from ArsR, such as a potential promoter here for YgbM.

### 3.5. Regulation of Transcription

Through our analysis, we noticed the abundance of 27 proteins increased when ArsR was present, consistent with an activating role of ArsR (Supplemental Table S4). We began a cursory exploration of the known transcription factors for the genes encoding these 27 proteins to identify trends that may highlight a conserved mechanism by which ArsR acts as an activator.

**Figure 7:**
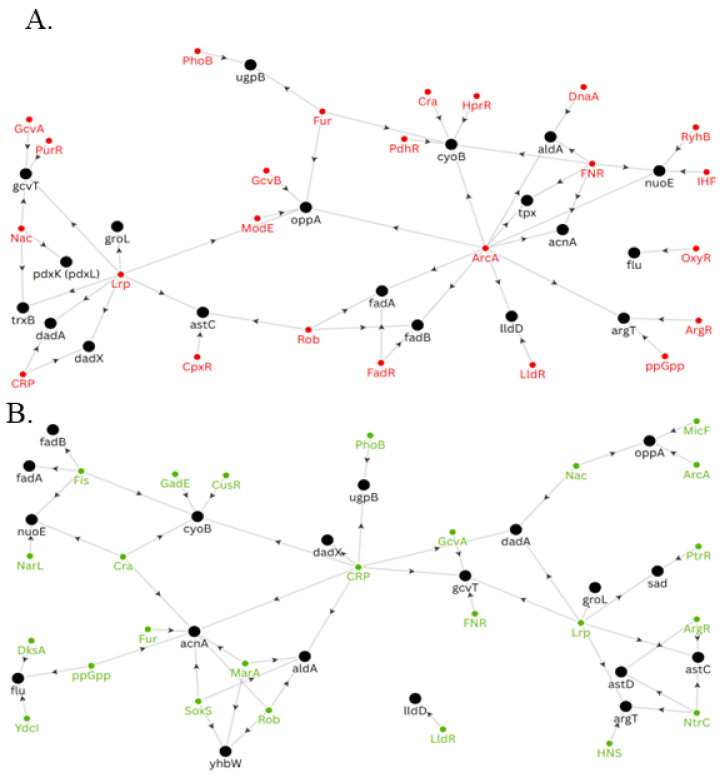
Regulators of identified proteins that fit the pattern of interest. Negative (**A**) and positive (**B**) gene regulatory networks, wherein black nodes represent genes, red nodes indicate inhibitory transcription factors, and green nodes show activating transcription factors. Some links appear in both networks (ex. oppA ← ArcA); this indicates transcription factors that have dual activating/inhibitory roles on specific genes. All the genes identified in these networks were found to be upregulated when ArsR was also more abundant.

In total, we identified 36 different transcription factors associated with 22 of the 27 genes using the RegulonDB and EcoCyc databases (Figure 7). Of these, 16 transcription factors affected at least two of the identified genes, and 20 affected only one gene. We next looked at the regulatory elements for each of the 36 transcription factors to further understand their regulatory network (Supplemental Figure S7). Together, these maps highlight the immense interconnectivity of this regulatory network, where ArsR may play a role. This data provides the framework for future exploration into the activation, repression, and/or coregulation that may be performed by ArsR. Additionally, this information serves as a reference for future findings pertaining to the regulation of any of the genes that exist along this regulatory network.

## 4. Conclusions

ArsR is the known repressor of the *ars* operons in microorganisms, which aid in cellular protection against arsenic stress. We have previously reported that the ArsR proteins in *A. tumefaciens,* which regulate the organism’s multiple *ars* operons, have broader regulatory effects that extend into other cellular processes. These seminal findings led to our investigation into *E. coli,* which lacks this arsenic-oxidizing ability and only has one *ars* operon. Our preliminary work in *E. coli* was focused solely on metalloenzymes, as the larger ArsR family of proteins are metalloregulatory transcription factors, which aid in maintaining metal homeostasis. The results of this study support and expand upon our previous work exploring how the presence of the functional and authentic ArsR in *E. coli* regulates the proteome beyond ArsRBC and metalloproteins. In our data, we saw that the absence of functional ArsR (e.g., AW3110 strain) consistently correlated with reduced abundance of multiple metabolic and redox-related proteins, while its presence (in the *arsR* complement and K-12 under As(III)-stressed conditions) coincided with increased abundance across diverse pathways.

We cannot rule out that slight genetic differences between the K-12 sub-strains MG1655 (K-12 wild type) and W3110 (AW3110 and the *arsR* complement) could impact protein abundance in these experiments [45]. These include systems linked to pyruvate and proline metabolism, and key components of thioredoxin redox buffering. Rather than repressing these systems or not being involved at all, the presence of ArsR appears necessary for their full induction under As(III) stress. This pattern positions ArsR not only as a gatekeeper of classical arsenic detoxification but also as a modulator, or even an enabler of broader metabolic plasticity. Given this finding, it is important to consider that we are basing this on protein abundance. This raises two potential limitations to the extension of our findings. First, transcriptomics and metabolomics data under the same conditions would most certainly add to our understanding of the functional role of ArsR. Much is discussed only based on proteomic differences, where metabolomics and even transcriptomics could have made a more complete assessment. Second, we did not take into consideration proteoforms or post-translational modifications. This deeper step of protein analysis would certainly clarify our understanding of the biology behind ArsR. That said, because the focus of this work is on ArsR as a transcriptional regulator, not tracking proteoforms is a relatively minor limitation because abundance is the key metric, not activity.

In summary, the data presented herein challenge the field to take a fresh look at the role of ArsR. A fitting example is uncharacterized protein YgbM, which is virtually absent in all groups except those with ArsR and significant As(III) stress, demonstrating ArsR’s role in activation of protein expression. The convergence of pathways feeding into the TCA cycle, generating glutamate and proline, and bolstering redox homeostasis in an ArsR-dependent manner suggests an integrated network. Here, ArsR may not merely lift repression in the presence of As(III) but instead coordinate an expanded transcriptional program to maintain redox balance, energy production, and cellular repair. These findings contribute to growing evidence that ArsR has a deep functional reach that extends well beyond the repression of *ars* genes and highlights its central, and potentially activating, role in orchestrating adaptive responses to arsenic-induced stress. This is important at least at two levels. First, ArsR was a founding member and model protein in the SmtB/ArsR family of transcriptional repressor proteins [1]. As such, it is reasonable to assume that other transcriptional repressors may similarly function in unpredicted regulatory patterns. Second, prior studies with the soil bacterium *A. tumefaciens* [10,11] laid down a foundational understanding of how ArsR has widespread regulatory effects that extend beyond the known *arsR*-regulated operon and arsenic resistance. Our other prior efforts [13] and the current study move these important discoveries into *E. coli*, which is an important member of the human gut microbiome [15] that is well recognized to be of significant importance to human health and welfare [46,47,48]. ArsR is widespread in the human gut microbiome [14] and thus even in the absence of a traditional ligand, As(III), one should expect it to exert regulatory effects.

## Data Availability

The original contributions presented in this study are included in the article and Supplementary Materials. The mass spectrometry proteomics data are available via ProteomeXchange with identifier PXD071635. Further inquiries can be directed to the corresponding author.

## Supplementary Materials

The following supporting information can be downloaded at https://www.mdpi.com/article/10.3390/proteomes14010001/s1: Figure S1: Experimental schematic and overview of statistical significance across proteome-wide comparisons; Figure S2: Principal component analysis of all the groups; Figure S3: L-alanine degradation is differentially regulated under arsenic stress; Figure S4: Differential expression of glutathione synthesis pathway proteins in response to arsenite exposure across *E. coli* strains; Figure S5: Strain-dependent expression of glutaredoxins involved in redox balancing under arsenite stress; Figure S6: Expression of glutathione synthetase and glutathione reductase in response to presence/absence of ArsR and or As(III) treatment; Figure S7. Regulation network of the transcription factors which regulate the proteins with the pattern of interest; Table S1: Proteins identified from shotgun proteomics; Table S2: Proteins that are significantly different in abundance using a Tukey’s HSD at an FDR < 0.001; Table S3: Cell counts and Cell Size; Table S4: Identified Proteins with the Pattern of Interest.
