# Supplementary material for: Beyond Repression: ArsR Functions as a Global Activator of Metabolic and Redox Responses in Escherichia coli
Source: Proteomes. 2026 Jan 4;14(1):1. doi: 10.3390/proteomes14010001 (PMC12821715; doi:10.3390/proteomes14010001)
Supplement: Supplementary file 1 [file proteomes-14-00001-s001.zip › 20251107_Supplelmental Figures.pdf]

Supplemental Methods

CME Methods – Orbitrap Eclipse

Protein samples were reduced, alkylated, and purified by chloroform/methanol extraction prior to digestion with sequencing grade modified porcine trypsin (Promega). Tryptic peptides were then separated by reverse phase XSelect CSH C18 2.5 um resin (Waters) on an in-line 150 x 0.075 mm column using an UltiMate 3000 RSLCnano system (Thermo). Peptides were eluted using a 60 min gradient from 98:2 to 65:35 buffer A:B ratio. Eluted peptides were ionized by electrospray (2.4 kV) followed by mass spectrometric analysis on an Orbitrap Eclipse Tribrid mass spectrometer (Thermo). MS data were acquired using the FTMS analyzer in profile mode at a resolution of 120,000 over a range of 375 to 1200 m/z. Following HCD activation, MS/MS data were acquired using the ion trap analyzer in centroid mode and normal mass range with a normalized collision energy of 30%. Proteins were identified by database search using MaxQuant (Max Planck Institute) with a parent ion tolerance of 3 ppm and a fragment ion tolerance of 0.5 Da. Scaffold Q+S (Proteome Software) was used to verify MS/MS based peptide and protein identifications. Protein identifications were accepted if they could be established with less than 1.0% false discovery and contained at least 2 identified peptides. Protein probabilities were assigned by the Protein Prophet algorithm [*Anal. Chem.* **75:** 4646-58 (2003)].

Buffer A = 0.1% formic acid, 0.5% acetonitrile
Buffer B = 0.1% formic acid, 99.9% acetonitrile

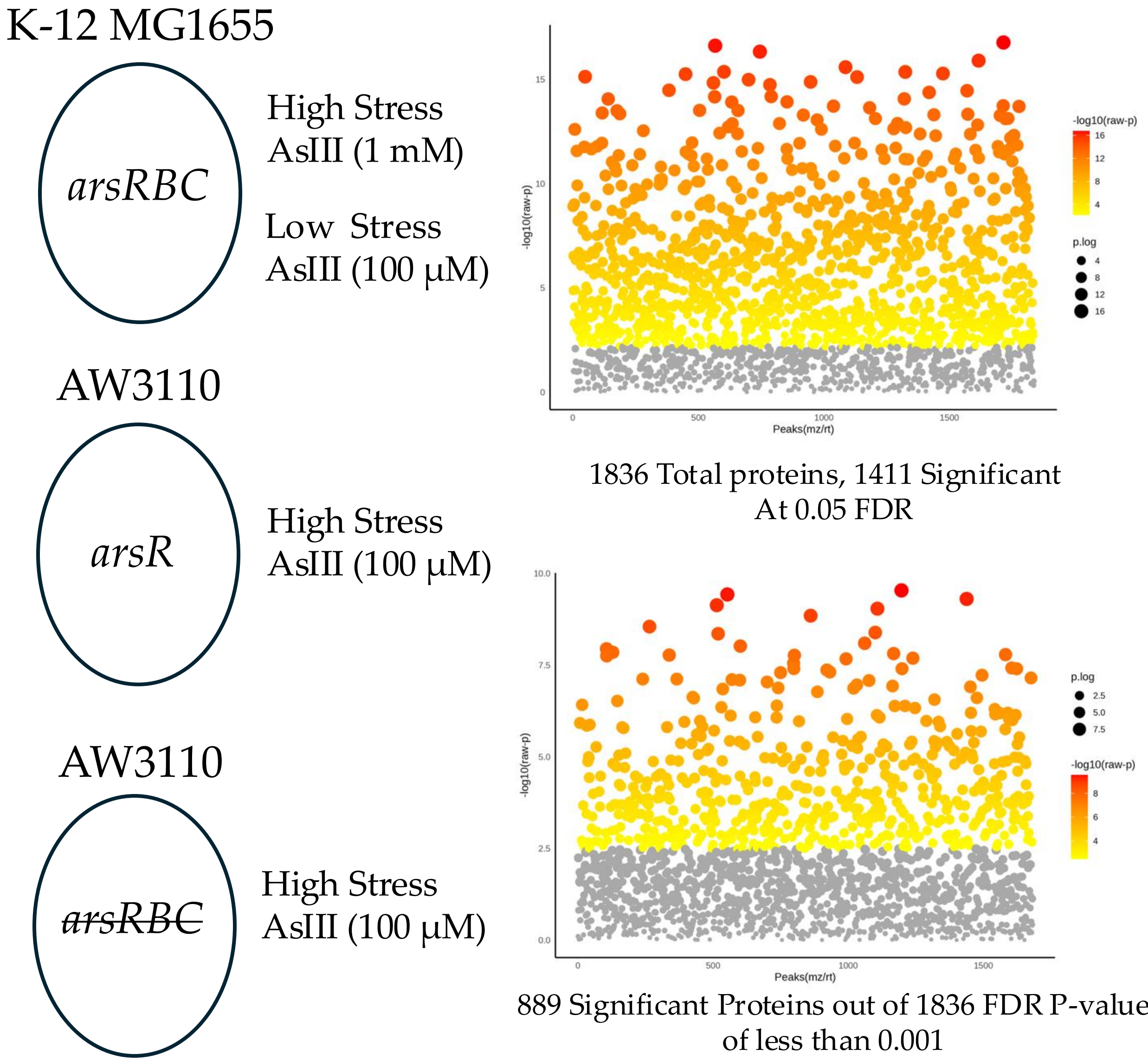

Supplemental Figure 1: Experimental schematic and overview of statistical significance across proteome-wide comparisons.

Left schematic is a representation of the three *E. coli* strains used to assess ArsR-dependent regulation. K-12 MG1655 contains the native *arsRBC* operon, AW3110 + *arsR* carries a plasmid borne *arsR* gene, and AW3110 which lacks the *arsRBC* operon. Each strain was subjected to both high arsenite stress and control conditions to evaluate global proteomic responses linked to ArsR presence. In addition, K-12 had an additional group to observe the same arsenite concentrations as the other two strains.

The right, upper panel displays the distribution of statistical significance across 1,836 quantified proteins, with adjusted p-values (FDR) plotted as –log10(p) values. A total of 1,411 proteins passed the significance threshold at a false discovery rate of 0.05. Data points are colored based on p-value magnitude (yellow to red gradient) and scaled by effect size as indicated by the dot size.

The right, lower panel refines this threshold, showing proteins meeting a stricter criterion (raw $p < 0.001$), highlighting 889 proteins as significantly differentially expressed. Similar color scaling and dot sizing represent relative p-value strength and magnitude. These results demonstrate a substantial shift in protein abundance across conditions, with many proteins exhibiting highly significant changes under the tested contrasts.

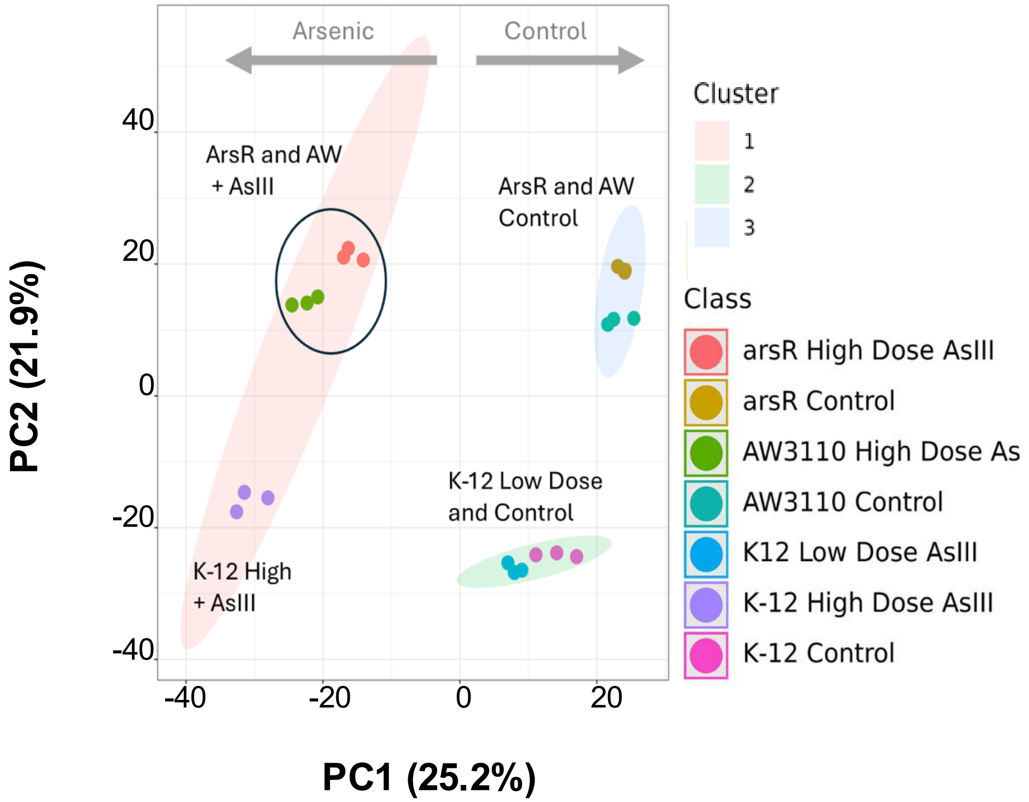

Supplemental Figure 2. Principal component analysis of all the groups (A) Principal component analysis (PCA) was applied to normalized proteomics data to explore global variation across strains and arsenite treatments. Points represent individual samples and are colored by treatment condition, while ellipses indicate groupings identified through K-means clustering. Three clusters are shown. **Cluster 1** (yellow) includes both AW3110 and arsR-complement samples exposed to 100 μM AsIII. **Cluster 2** (blue) contains K-12 controls and the 100 μM AsIII-treated K-12 group. **Cluster 3** (green) includes untreated AW3110 and arsR-complement samples. K-12 samples treated with 1 mM AsIII form a separate subgroup within Cluster 1.

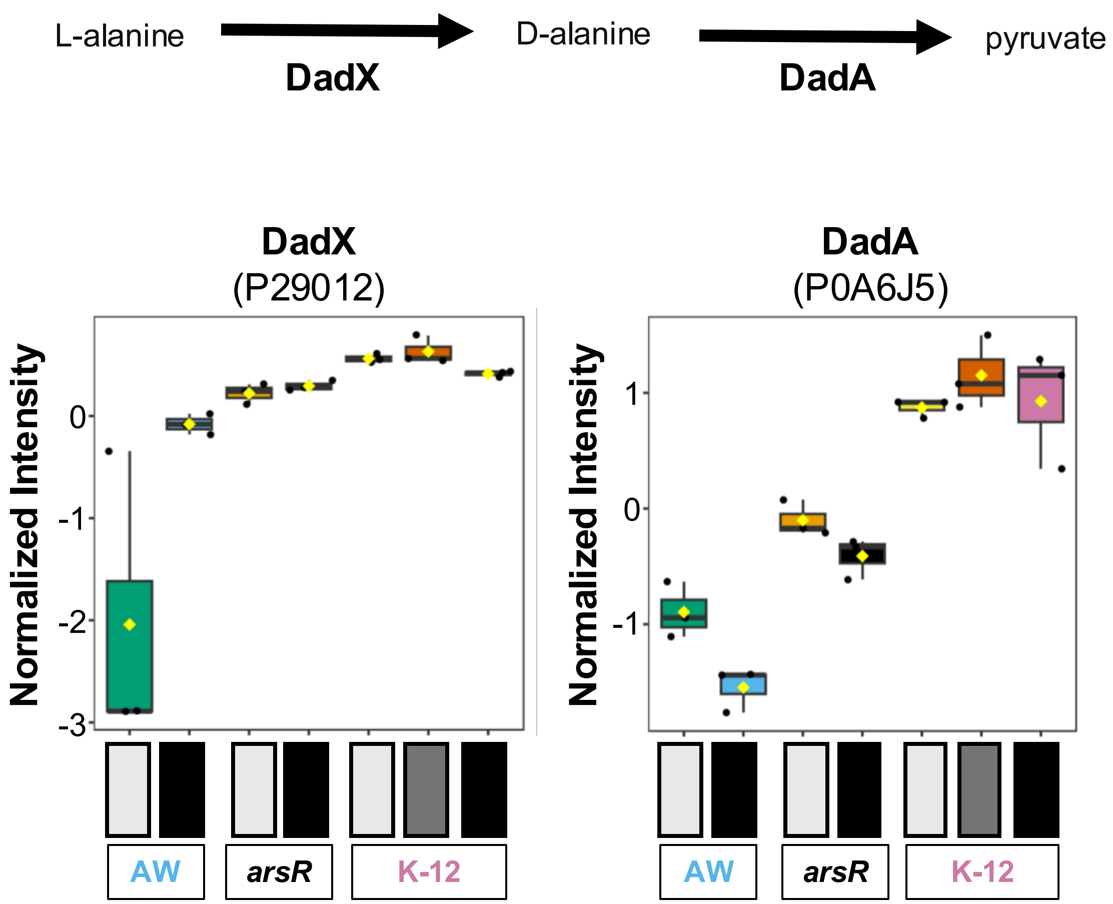

Supplemental Figure 3: L-alanine degradation is differentially regulated under arsenic stress. Schematic showing the L-alanine degradation pathway through DadX and DadA.. DadX catalyzes the conversion of L-alanine to D-alanine, and DadA further deaminates D-alanine to pyruvate. Normalized intensity plots of DadX (P29012) and DadA (P0A6J5) under no AsIII (white), low AsIII (gray), and high AsIII (black) conditions across the three *E. coli* strains. The strains are represented by either AW for AW3110, *arsR* for the *arsR* complemented strain, or K-12.

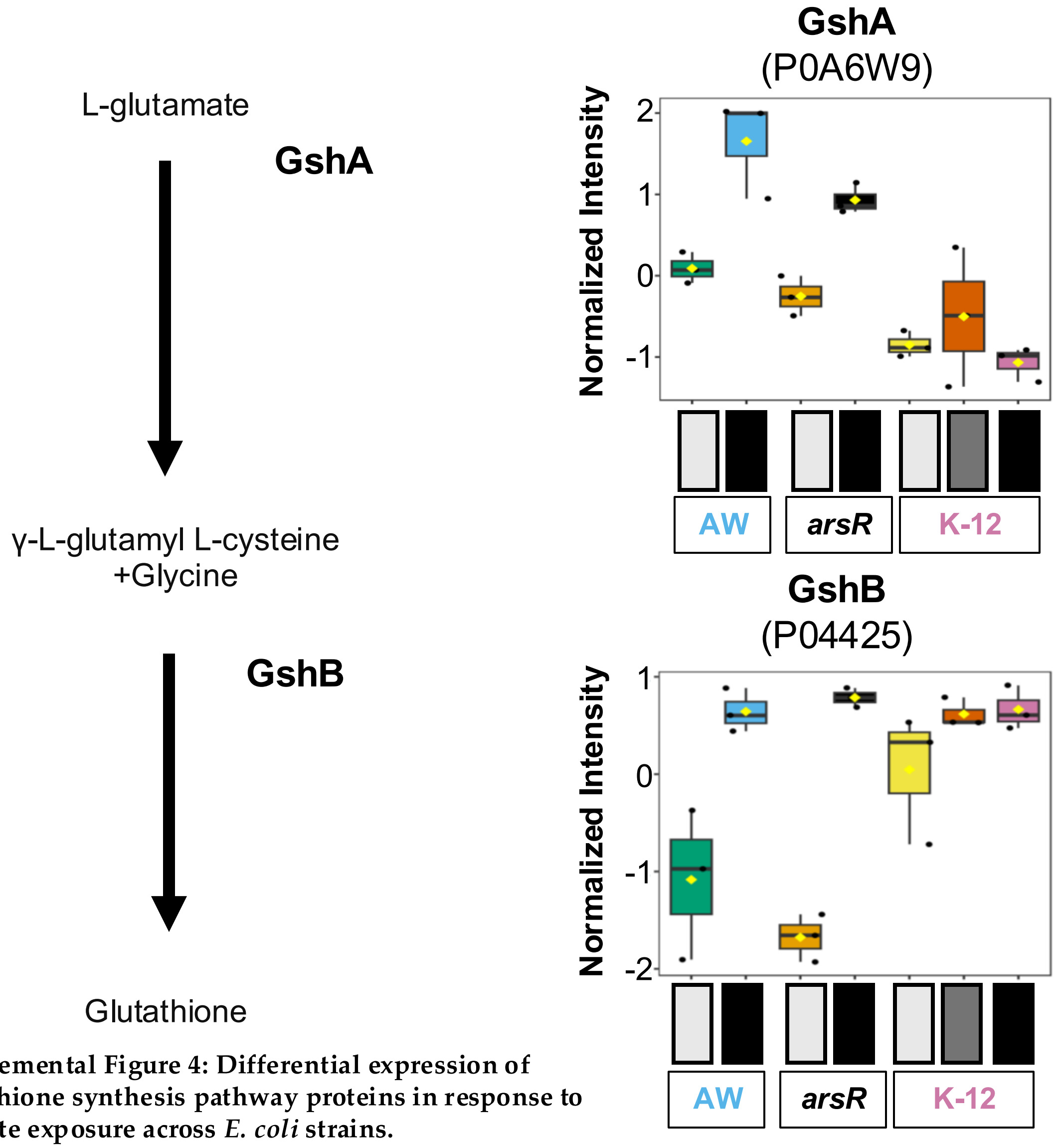

Supplemental Figure 4: Differential expression of glutathione synthesis pathway proteins in response to arsenite exposure across *E. coli* strains.
Glutathione biosynthesis enzymes **GshA** (P0A6W9) and **GshB** (P04425). On the right, ANOVA-normalized expression levels are displayed by condition: light gray for control, gray for low arsenic, and black for high arsenic exposure. The strains are represented by either AW for AW3110, *arsR* for the *arsR* complemented strain, or K-12.

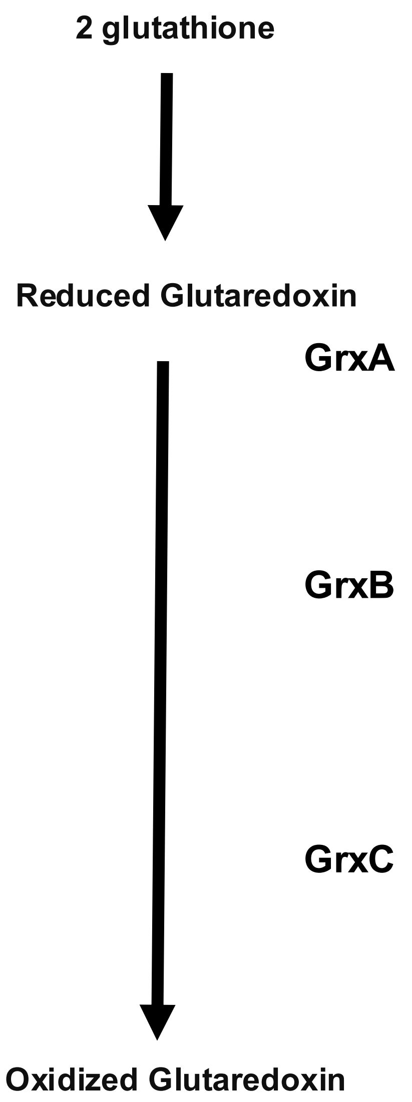

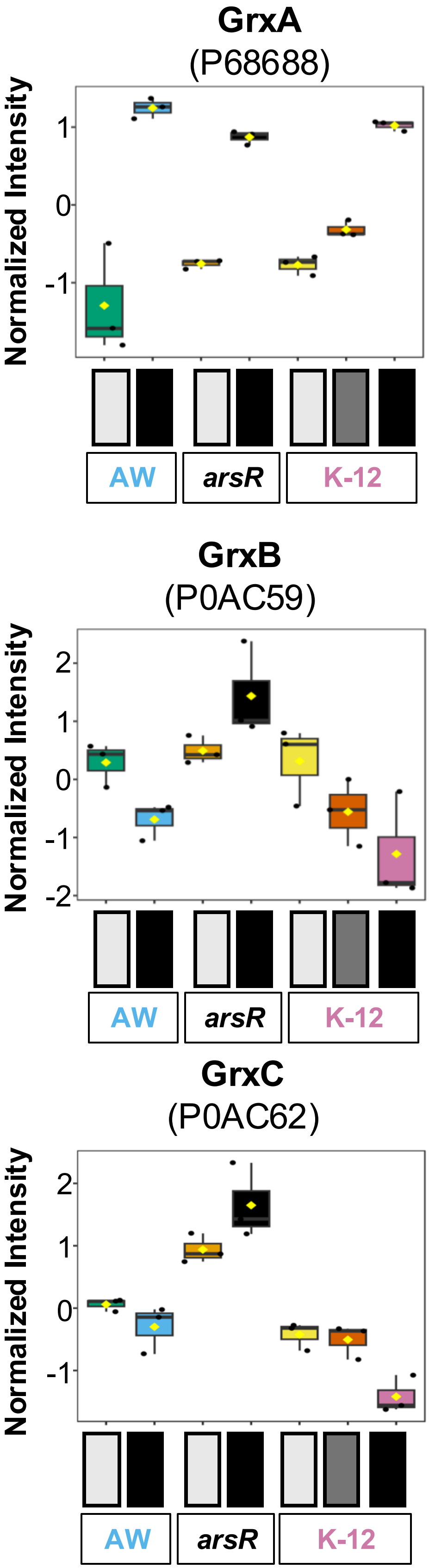

Supplemental Figure 5: Strain-dependent expression of glutaredoxins involved in redox balancing under arsenite stress.
This figure presents relative expression levels of three glutaredoxin proteins (**GrxA** (P68688), **GrxB** (P0AC59), and **GrxC** (P0AC62)). These three proteins are involved in thiol-disulfide exchange reactions utilizing glutathione as a reducing agent. One the right is the ANOVA normalized intensity relative abundance plots of each of the represented proteins where the gradient represents either control (light grey), low dose arsenic (grey), or high dose arsenic (black). The strains are represented by either AW for AW3110, *arsR* for the *arsR* complemented strain, or K-12.

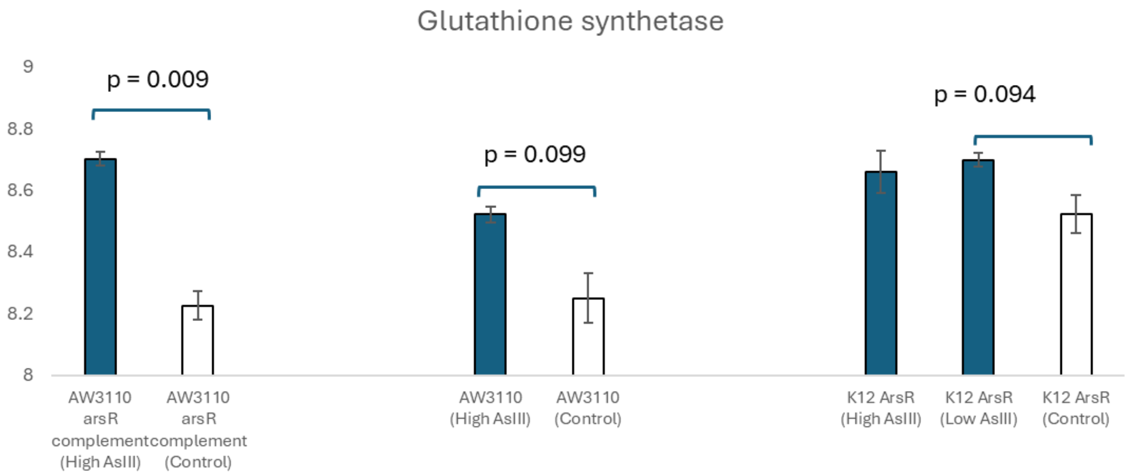

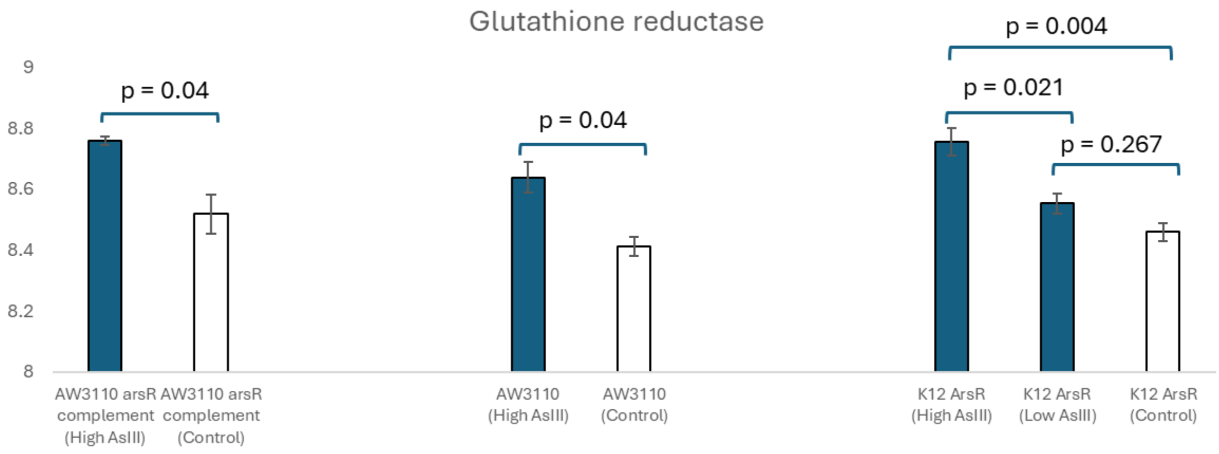

Supplemental Figure 6: Expression of glutathione synthetase and glutathione reductase in response to presence/absence of ArsR and or As(III) treatment. Data normalized intensities are log10 transformed (represented on the y-axis) and illustrate mean ± SE of three replicate cultures normalized for total protein per sample. p-values indicate statistical tests of the paired t-test for each pair(s) grouped based on As(III) treatment.

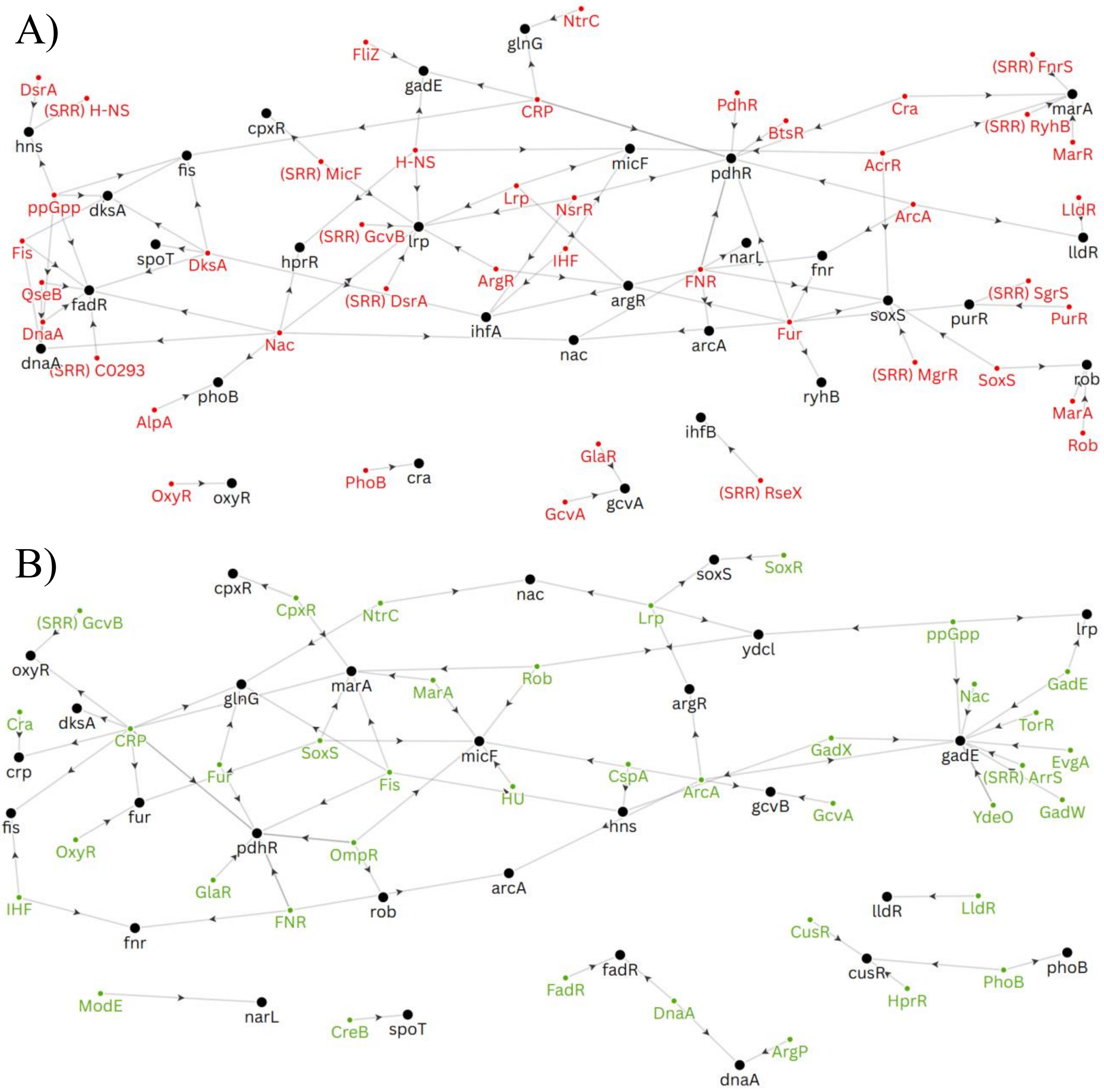

Supplemental Figure 7. Regulation network of the transcription factors which regulate the proteins with the pattern of interest. These diagrams show positive (A) and negative (B) gene regulatory networks, wherein black nodes represent genes, red nodes indicate inhibitory transcription factors, and green nodes show activating transcription factors. There are some links that appear in both networks (ex. argR ← Lrp); this indicates transcription factors that have dual activating/inhibitory roles on specific genes. The genes included in these networks encode the transcription factors that influence the genes which were found to be up regulated when ArsR was also upregulated (Figure 7)
